# *Trypanososma brucei rhodesiense* Sleeping Sickness, Uganda

**DOI:** 10.3201/eid1810.111213

**Published:** 2012-10

**Authors:** Lea Berrang-Ford, Charles Wamboga, Abbas S.L. Kakembo

**Affiliations:** McGill University, Montreal, Quebec, Canada (L. Berrang-Ford);; and Ministry of Health, Kampala, Uganda (C. Wamboga, A.S.L. Kakembo)

**Keywords:** human African trypanosomiasis, Trypanosoma brucei rhodesiense, parasites, sleeping sickness, Africa, spatial distribution, epidemiology, Uganda

**To the Editor:** The past 2 decades have heralded notable success in efforts to control sleeping sickness (human African trypanosomiasis [HAT]) in Africa. HAT is a neglected tropical disease with major public health and economic effects in sub-Saharan Africa, and its effects on livestock productivity and development are considered major constraints to alleviating poverty in this region (*1*,*2*). Because of concerted and coordinated continental control efforts, its incidence has steadily decreased.

Despite these successes, concern has increased recently regarding potential convergence of the 2 causes of HAT (*Trypanosoma brucei gambiense* and *T. brucei rhodesiense*). These organisms differ in transmission and how infections are diagnosed and treated, and control, and have never coincided in the same area. Uganda is the only country with endemic distributions of these 2 trypanosome species, and convergence there represents a major public health concern, given the potential for overlapping infections to compromise treatment and control programs and spread into neighboring countries (*3*,*4*).

Risk for convergence led to an international emergency intervention. In 2006, an international public–private partnership, Stamp Out Sleeping Sickness (SOS), was established to control spread of this disease in central Uganda (*5*). However, despite the continental effect of convergence of the 2 causes of HAT, little is known about trends in incidence and epidemiology of HAT in central Uganda. We report results of data analysis for HAT caused by *T. b. rhodesiense* during 2000–2009.

This study was approved by the ethics review board for human subjects at McGill University (Montreal. Quebec, Canada). We obtained data on case-patients given a diagnosis of *T. b. rhodesiense* HAT at a HAT treatment unit in Uganda. These diagnoses were reported to the National Sleeping Sickness Control Program of the Ministry of Health (*4*,*6*). Data were assigned locations by parish, and analyses focused on spatiotemporal trends in case occurrence. The final cleaned dataset contained 2,501 reported cases of presumed *T. b. rhodesiense* HAT.

In the past 10 years in Uganda, 140 cases of fatal *T. b. rhodesiense* HAT have been reported. However, given estimates of underreporting and cessation of active surveillance, actual deaths are likely > 1,700 (170 deaths/year) (*6*). Notably, mortality rates have increased from an average of 5% in the early 2000s to ≈10% in later years, and rates have been higher in recently affected districts. This pattern is predominantly driven by higher mortality rates in newly affected SOS districts in central Uganda, in which diagnostic and treatment delays are higher, and from which an increasing proportion of HAT cases are originating.

Patients in SOS intervention districts were more likely to report cases in late stages of the disease (p<0.01, by χ^2^ test). The mortality rate was >3-fold higher for persons with late-stage cases (8.1%) than for those with early-stage cases (2.4%) (p<0.01, by χ^2^ test). Given that central Uganda is the critical zone for convergence and intervention, such evidence of presumed diagnosis and treatment delay is cause for concern.

The SOS phase 1 intervention period (2006–2008) coincided with a period of reduced reported prevalence of HAT (Figure). The monthly average was 27 cases/month before the intervention and 10 cases/month after the intervention (p<0.01, by Mann-Whitney test). However, a substantive component of reduction in incidence occurred in districts not included in the SOS intervention program. This pattern may reflect reporting bias caused by a transition in Uganda in 2005 to a period of passive surveillance and underfunding for national reporting. Therefore, it remains unclear to what extent increased international attention and SOS intervention have contributed to HAT prevention and control. The absence of a clear increase in incidence after reinstatement of national data acquisition in 2008 provides an early indication that interventions may be contributing to the decrease in, or at least to stabilization of, geographic spread in central Uganda.

**Figure:**
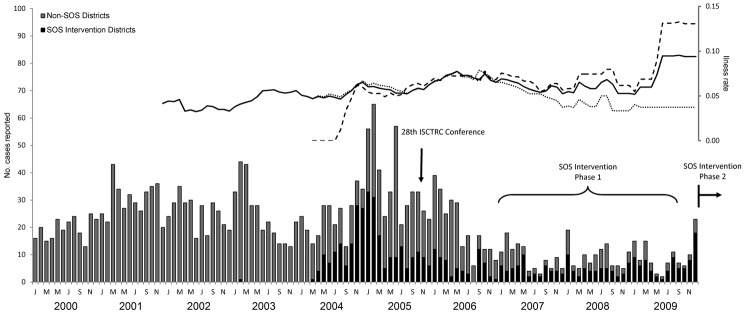
Human African trypanosomiasis cases and deaths by month, Uganda, 2000–2009. Bars indicate cases in districts in the Stamp Out Sleeping Sickness (SOS) intervention region and outside the SOS region. Solid line indicates overall 24-month moving average of deaths, dashed line indicates 24-month moving average of deaths in SOS intervention districts, and dotted line indicates 24-month moving average of deaths in non-SOS districts. ISCTRC, International Scientific Council for Trypanosomiasis Research and Control; J M M J S N, Jan, Mar, May, Jul, Sep, Nov.

HAT data indicate seasonality of this disease; incidence is higher during January, February, and March (p = 0.04, by Mann-Whitney test). Seasonality of HAT incidence has been noted elsewhere and linked to seasonal influences on tsetse habitat suitability. We propose that seasonality of cattle trading may also play a role because cattle purchases increase before the Christmas season, which promote pathogen spread and increased transmission. This finding is consistent with research highlighting the role of livestock markets in the spread of *T. b. rhodesiense* in central Uganda and would further support a body of literature suggesting, as espoused by the SOS initiative, that control of animal reservoirs of the disease is a critical component of intervention measures (*2*,*7*–*9*). Implementation and enforcement of regulations for treatment of cattle before sale at markets would also contribute to limiting spread (*9*,*10*);

Interventions in districts in central Uganda in which convergence is predicted have been slow and incomplete. If convergence has occurred, this finding indicates that a specific region in Africa has had concurrent infection with both causes of HAT, with implications for prevention, treatment, and control. Since 2000, Uganda has had continued northward spread of *T. b. rhodesiense* infections, reducing the distance with TbG to <100 km, which we believe is a conservative estimate. Reinstatement of active surveillance of HAT and support for central data collection in Uganda are long overdo and warranted immediately.
